# Does this patient have pheochromocytoma? A systematic review of clinical signs and symptoms

**DOI:** 10.1186/s40200-016-0230-1

**Published:** 2016-03-31

**Authors:** M. Pourian, Davani B. Mostafazadeh, A. Soltani

**Affiliations:** 1 Endocrinology and Metabolism Research Center, Endocrinology and Metabolism Clinical Sciences Institute, Tehran University of Medical Sciences, Tehran, Iran; 2 Evidence based Practice Research Center, Endocrinology and Metabolism Research Center, Tehran University of Medical Sciences, Tehran, Iran

**Keywords:** Pheochromocytoma, Likelihood ratio, Clinical exam, Sensitivity, Specificity

## Abstract

**Context:**

Pheochromocytoma is a rare disease but with high mortality if it is not being diagnosed early. Several biochemical tests with high accuracy have been obtained, but the clinical threshold for request of these tests is not determined clearly.

**Objectives:**

To determine the Likelihood Ratios of clinical symptoms and signs in diagnosing pheochromocytoma. And also meta-analysis of their sensitivity in this disease.

**Data sources:**

MEDLINE was searched for relevant English-language articles dated 1960 to February 2014. Bibliographies were searched to find additional articles.

**Study selection:**

We included original studies describing the sensitivity and/or likelihood ratios of signs and symptoms in clinical suspicion of pheochromocytoma. Their method of diagnosis should have been based on pathology. We excluded specific subtypes or syndromes related to pheochromocytoma, or specific ages or gender. Also we excluded studies before 1993 (JNC5) which no definition of hypertension was presented. 37 articles were chosen finally.

**Data extraction:**

Two authors reviewed data from articles independently and gave discrepancies to third author for decision. The aim was extraction of raw numbers of patients having defined signs or symptoms, and draw 2 × 2 tables if data available. We meta-analyzed sensitivities by Statsdirect and Likelihood Ratios by Meta-disc soft wares. Because our data was heterogeneous based on I^2^ > 50 % (except negative Likelihood ratio of hypertension), we used random effect model for doing meta-analysis. We checked publication bias by drawing Funnel plot for each sign/symptom, and also Egger test.

**Data synthesis:**

The most prevalent signs and symptoms reported were hypertension (pooled sensitivity of 80.7 %), headache (pooled sensitivity of 60.4 %), palpitation (pooled sensitivity of 59.3 %) and diaphoresis (pooled sensitivity of 52.4 %). The definition of orthostatic hypotension was different among studies. The sensitivity was 23–50 %.

Paroxysmal hypertension, chest pain, flushing, and weakness were the signs/symptoms which had publication bias based on Funnel plot and Egger test (*P* value < 0.05). Seven of the articles had control group, and could be used for calculating LR of signs/symptoms. Diaphoresis (LR+ 2.2, LR- 0.45), Palpitation (LR+ 1.9, LR- 0.52) and headache (LR+ 1.6, LR- 0.24) were significant symptoms in clinical diagnosis of pheochromocytoma. Other signs and symptoms had been reported in only one study and could not have been meta-analyzed. Classic triad of headache, palpitation and diaphoresis in hypertensive patients had the LR+ 6.312 (95 % CI 0.217–183.217) and LR- 0.139 (95 % CI 0.059–0.331). Surprisingly, hypertension was not important in clinical suspicion of pheochromocytoma, and even normotension increased the probability of the disease.

**Conclusions:**

By available data, there is no single clinical finding that has significant value in diagnosis or excluding pheochromocytoma. Combination of certain symptoms, signs and para-clinical exams is more valuable for physicians. Further studies should be done, to specify the value of clinical findings. Until that time the process of diagnosis will be based on clinical suspicion and lab tests followed by related imaging.

## Clinical scenario

### Case 1

A 35 year old woman was referred by her family physician because of recurrent spells of headache, dizziness, and sweating since 6 months ago. She had also experienced dyspnea and palpitation followed by chest discomfort. Each time, she was admitted to the hospital with high blood pressure and heart rate. But the physical exam between attacks was normal.

### Case 2

A 50 year-old man came to his family physician with moderate bitemporal headache. On physical exam, his blood pressure was 170/100 mmHg and pulse rate was 70. He had no chest pain, dyspnea or blurred vision. He had experienced such headaches in the last 6 months about once a month.

## Background

Pheochromocytoma is a rare tumor with an annual incidence of 1–4/10^6^ population [1]. It is popular for causing hypertension; however, It is an uncommon cause of hypertension, estimated to occur in approximately 0.1 to 1 % of hypertensive patients [1, 2, 4, 5]. It is suggested that most doctors meet only one patient with pheochromocytoma in their working lifetime and a large general hospital admits -on average- one such patient annually [4]. Despite the low frequency, pheochromocytoma is fascinating and challenging to clinicians because it has lethal potential if untreated, and possible long term cure -in the majority- if diagnosed and treated surgically. Clinical awareness of this tumor should be stressed because 1) Surgical removal is curative in more than 90 % of patients (The 5-year patient survival after removal of benign pheochromocytoma has been ranged from 84 to 96 %) [6], 2) Tumor excision has significant effect on hypertension, the most important cause of pheochromocytoma related mortality and morbidity. In the follow up of surgeries, it has been shown that about 60 % of patients became normotensive [4, 7–9]. In patients with persistent hypertension after surgery, the mean arterial pressure decreased significantly [7] and was controlled better with anti-hypertensive drugs [10]; hypertensive crises disappeared after surgery [10, 11]; and hypertension-related complications regressed significantly [11], 3) Biochemical testing and imaging together have high accuracy in detection of the disease 4) Some drugs and in particular, anesthetic agents may potentiate the life-threatening effects on the heart and circulation of catecholamines secreted by this tumor, and 5) If it is left untreated, fatal complications often ensue, most of which are related to hypertension (In a series of autopsy from the Mayo Clinic that spanned 50 years, 75 % of the cases were undiagnosed during life, although they were symptomatic) [12]. Thus, early clinical diagnosis of pheochromocytoma is imperative, to allow clinicians to efficiently complete further investigations and initiate appropriate treatment with the goal of minimizing morbidity and mortality.

P.F. Plouin studied 2585 hypertensive patients to find out the value of clinical triad –headache, palpitation and diaphoresis, in the diagnosis of pheochromocytoma [13]. He found positive Likelihood ratio (LR) of 14.6 and negative likelihood ratio of 0.1 for the triad of symptoms. It showed that clinical picture can predict the probability of the pheochromocytoma to a good level. From that time, this triad became the base of clinical suspicion for endocrinologists to further work up to detect pheochromocytoma. But pheochromocytoma shows many other symptoms and signs which may have additional benefits for clinical diagnosis. We did a systematic review in order to define the value of each piece of the clinical picture to identify patients with pheochromocytoma whom further diagnostic tests are indicated.

## Pathophysiology and clinical presentation of pheochromocytoma

First described in 1886 by Fränkel, pheochromocytomas are tumors derived from the chromaffin cells of the embryonic neural crest [1]. Chromaffin cells are post-ganglionic sympathetic neurons which produce catecholamines. When fresh tissue samples are oxidized with certain fixatives, their catecholamine content is stained dark grey-brown (“*peso*” in Greek). These cells are mainly located in the adrenal medulla (in fact, approximately 85–95 % of pheochromocytomas are located in the adrenal medulla) [2, 3]. Tumors arising from extra-adrenal chromaffin cells are termed paragangliomas and they can be found along the paravertebral and para-aortic axes (sympathetic paraganglia have a neck-to-pelvis distribution, while parasympathetic paraganglia are found almost exclusively in the neck and skull base, along the branches of glossopharyngeal and vagus nerve).

**Fig. 1:**
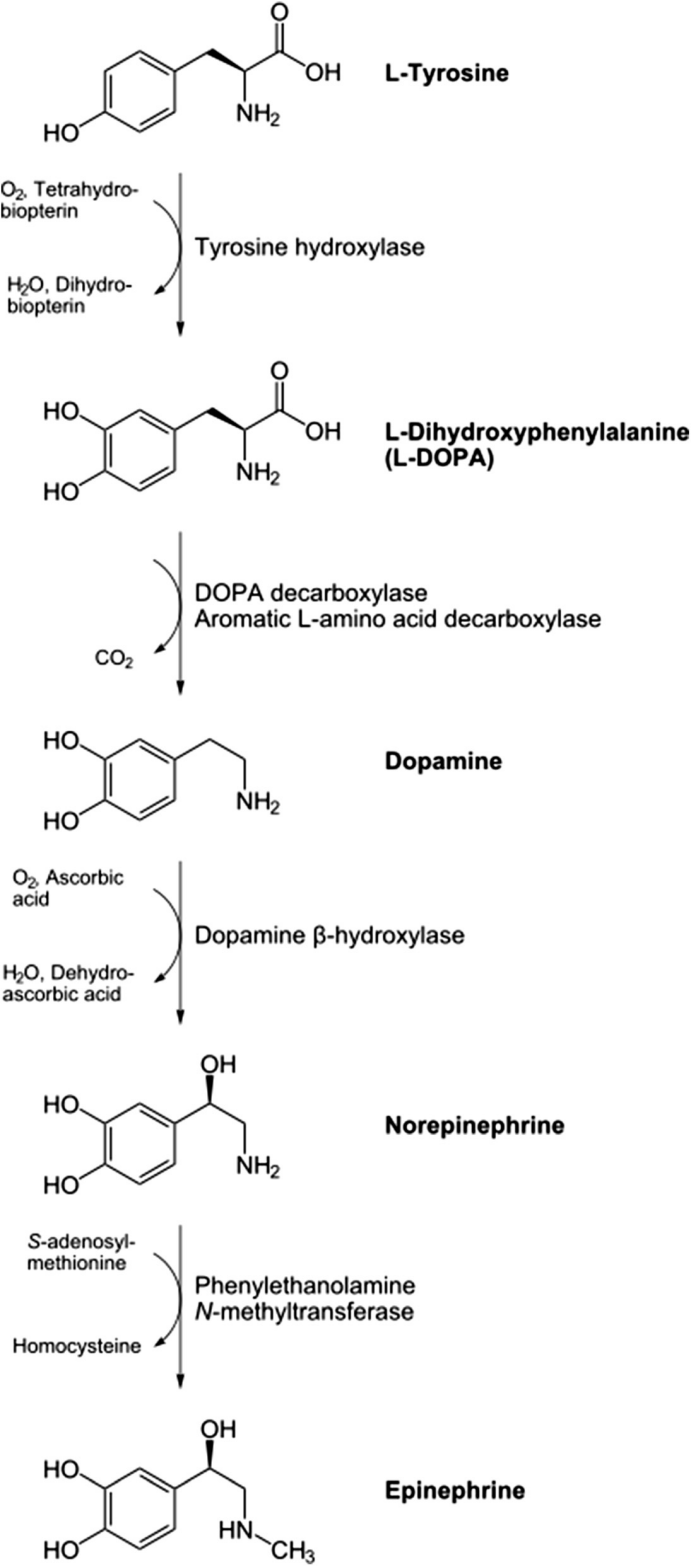
The catecholamine metabolism in normal human cells

Although about 4 % of adrenal masses incidentally found are known to be pheochromocytoma [2], this tumor is popular for its catecholamine secretion, and symptoms the catecholamines produce. The most catecholamines secreted are epinephrine, norepinephrine and dopamine. Figure 1 illustrates the catecholamine metabolism in normal human cells. Normal adrenal glands contain mostly epinephrine. Most pheochromocytomas secrete predominantly norepinephrine; and about 15 % secrete predominantly epinephrine. Dopamine β-hydroxylase, responsible for converting dopamine to norepinephrine, may be missing in immature tumors; Thus, the presence of a tumor secreting predominantly dopamine indicates a higher probability of malignancy. Pheochromocytoma has been called the “Great Mimic” since its manifestations can resemble so many other conditions, which may confuse clinicians [14]. The clinical presentation varies, ranging from an adrenal incidentaloma to hypertensive crises with associated cerebrovascular or cardiac complications [15]. The vast majority of symptoms and signs are attributable to the excess of catecholamines released by tumors continuously or paroxysmally. The most leading catecholamine-related sign for clinicians to suspect pheochromocytoma is hypertension. Related to hypertension, four patterns of blood pressure are seen. Sustained hypertension, paroxysmal hypertension, sustained hypertension with paroxysms, and normotension. This variation is somehow related to the catecholamine predominantly secreted by the tumor. The catecholamines exhibit different effects on different catecholamine receptors; typically, norepinephrine-mediated stimulation of α-receptors leads to vasoconstriction whereas epinephrine stimulates β_2_-receptors, causing vasodilatation. Subjects with predominantly norepinephrine-secreting pheochromocytoma (noradrenergic phenotype) develop sustained hypertension more frequently than subjects with predominantly epinephrine-producing pheochromocytomas (adrenergic phenotype) who present more often with paroxysmal symptoms. Dopamine-producing tumors often present with normotension [14]. Paroxysmal release of catecholamines constitutes the characteristic classic triad of episodic headache, sweating, and palpitations which is known as “an attack”. In some patients, a particular stimulus triggers an attack. Anesthesia and tumor manipulation are the most well-known triggers for catecholaminergic crisis; positional change, exercise, and various medications (e.g. TCAs, opiates, metoclopramide and radiographic contrast agents) are other possible precipitating factors. In others, no clearly defined precipitating event can be found, and the episodes occur in a random pattern.

In addition to the classic triad, patients often experience other symptoms such as anxiety, dyspnea, chest, abdominal or flank pain, nausea and vomiting, tremor, flushing, dizziness, visual symptoms such as blurred vision, and paresthesia. On the other hand, persistent vasoconstriction in patients with pheochromocytoma declines the blood volume leading to orthostatic hypotension [16]. The sudden out-pouring of epinephrine has been postulated as causing an elevation in body temperature by a combination of inducing hypermetabolism and impairing heat dissipation as a consequence of cutaneous vasoconstriction [17] (the leading cause of pallor during attacks). Hypermetabolism caused by catecholamines can cause weakness, fatigue and weight loss. Paradoxically, some patients have diarrhea, whereas others may have constipation.

Although chronic hypertension can cause cardiovascular complications, catecholamines directly have toxic effect on myocardium as well. During attacks different ECG changes evolve, which are resolved after the surgery. However, chronic exposure to catecholamine can lead to irreversible myocardial fibrosis [16].

## Methods

### Literature search

We reviewed Medline database from 1960 to February 2014 by the structured search strategy including both text word and MeSH term of the following keywords: pheochromocytoma, diagnosis, physical examination, medical history taking, sensitivity and specificity, reproducibility of results, observer variation and predictive value of tests; We limited the results to English language and humans^1^. The results were reviewed by two of the authors for relevance and quality. The aim was looking for original articles which reported the sensitivity and/or specificity of symptoms in patients with pheochromocytoma. Then, the results were discussed and the papers which the authors had disagreement, were given to the third author for decision about inclusion. We chose the papers which their method for disease-confirmation was based on histopathology and the data was extracted before diagnosis of the disease (in order to resolve recall bias). We excluded papers which studied a specific subtype of pheochromocytoma (e.g. malignant or familial), or just syndromes that pheochromocytoma was a part of them (e.g. MEN, Von Hippel Lindau disease). Also, we excluded the studies on only specific age/gender. Moreover, we reviewed the papers studying the value of biochemical testing in diagnosis of pheochromocytoma (we thought that these studies more possibly have specificity of symptoms beside their sensitivity, because of having control group); if they had clinical data of the patients, and if they had no clinical relevant data, we sent an email to the authors and asked for their clinical data if available. On the other hand, we tracked the references of review articles to find more original articles. In addition to electronic search, we did hand-searching using endocrinology textbooks.

### Definitions

We defined hypertension as blood pressure higher than 140/90 mmHg. As this definition was accepted in the fifth Joint National Committee on Detection, Evaluation, and Treatment of High Blood Pressure (JNC V) in 1993 [18], we sent emails to the authors of articles published before this time with no definition for hypertension in the article and asked if their definition was the same, and if we got no response, we ommited the article from analysis. Orthostatic hypotension was defined as a drop in systolic (20 mmHg) or diastolic (10 mmHg) blood pressure within 3 min of standing. Finally, because the definitions were different among the studies which checked this sign, we separately discussed the articles in the result section.

### Data analysis

After selection of articles for analysis, considering inclusion and exclusion criteria, we extracted the crude numbers of patients having symptoms or signs of possible pheochromocytoma. The symptoms consist of headache, palpitation and diaphoresis (three parts of the classic triad), total classic triad, flushing, palor, nausea/vomiting, weakness/fatigue, diarrhea, constipation, dizziness/vertigo, chest pain, abdominal pain, flank pain, dyspnea, paresthesias, anxiety, visual symptoms, and tremor. The signs we looked for, were hypertension and orthostatic hypotension. We filled the 2 × 2 tables for calculating LR for the symptoms that we could, and put the sensitivities together in a separate table. Meta-analysis was done if possible (number of studies more than one) for LR of symptoms and signs, and a meta-analysis was performed for sensitivities separately. Several factors affect distribution of symptoms and signs in studies, such as distribution of genders, malignant or benign disease and size of the tumor, but none of the studies had separated these factors (so we could not do subgroup analysis or meta-regression if the data was heterogeneous). We calculated heterogeneity by drawing Forest plot and I^2^ test. We considered heterogeneity as I^2^ > 50 %. Because our data was heterogeneous based on I^2^ (except one), we used random effect model for doing meta-analysis. We checked publication bias by drawing Funnel plot for each sign/symptom, and also Egger test. If the P value in Egger test is below 0.05, then we considered the data to have publication bias. Analysis of the sensitivity was done by Stats-direct software and analysis of the LRs was done by meta-disc software.

## Result

**Fig. 2:**
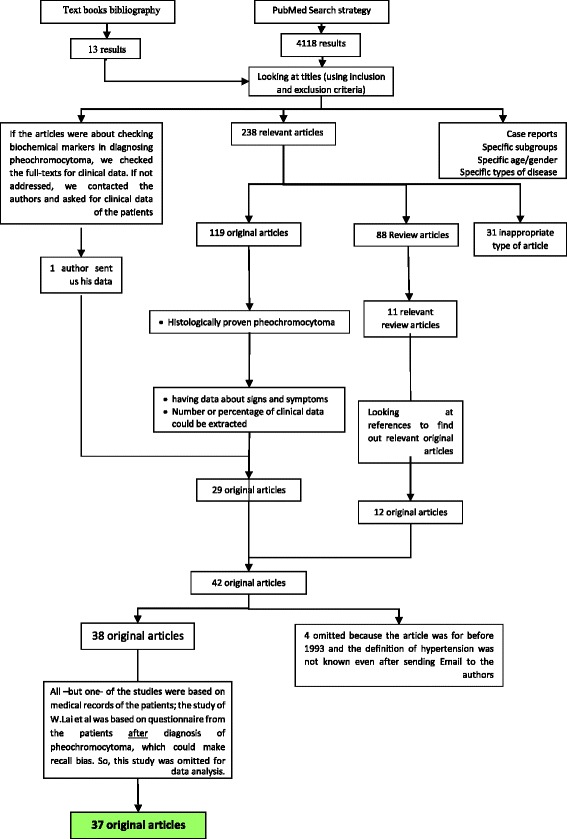
Systematic review flow diagram

The initial search strategy yielded 4118 results. And the result of our hand-searching was 13. Based on titles, 238 articles were selected. From the articles which had studied biochemical diagnosis of pheochromocytoma and we had sent emails to authors for clinical data, one article was received (ref 23). From these 238 articles, 31 were inappropriate type by abstract, 119 original and 88 review articles were extracted. After getting full-text for checking relevancy and quality analysis, and also considering inclusion and exclusion criteria for original articles, 29 were selected for data extraction. By reference-tracking, 12 more original articles were found. Totally 42 articles were considered for analysis. Based on our definition for hypertension, 4 articles were excluded because they were done before 1993, and the definition of hypertension was not specified, and there was no answer to our email for definition. All –but one- of the studies were based on medical records of the patients; the study of W. Lai et al. [19] was based on questionnaire from the patients *after* diagnosis of pheochromocytoma, which could make recall bias; so, this study was excluded from data analysis. Finally, 37 articles were analyzed (Fig. 2).

The characteristics of the articles are shown in Tables 1 and 2. Seven of these articles had control groups; five of which the control groups were the patients with suspected but excluded pheochromocytoma surgically or by follow-up, and in two others, the control group was hypertensive patients. In addition, in these two articles, the total population was hypertensive patients not the general population. So, data analysis of these two was done separately from the other five.

**Table 1:** Studies Assessing Clinical Presentations: studies without control group

Source	Clinical setting, Years	No. of Patients	Age, Mean (Range)	Gender (M, F)	Ref
Chung-Yau Lo et al., 2000	Queen Mary hospital, Hong Kong, China, 1981–1998	29 (24 diagnosed in life, 4 postmortem, 1 operated with an unexpected diagnosis	Median = 50 (18–75)	11,18	[4]
M. Mannelli et al., 1999	18 centers from all over Italy, 1978–1997	258	44 (8–84) in female	---	[7]
			46(12–79) in male		
Gennaro Favia et al., 1998	A university endocrine surgery unit, Italy, 1977-1996	55	41 (10–63)	28,27	[8]
Gunnar Stenstrom et al., 1988	A university hospital, Sweden, 1956–1982	64	45 (15–79)	30,34	[9]
J.W. Lance and Hinterberger, 1976	A University Hospital in Sydney, Australia	27	9–54	14,13	[15]
Patócs et al., 2004	Faculty of Medicine, Semmelweis University in Budapest, Hungary. 1995–2003	41	(20–73)	11,30	[21]
FC Hernandez et al., 2000	University Hospital “Virgen de la Arrixaca”, Murcia, Spain. 1994–1998	57	42 (7–71)	34,23	[22]
Laurence Amar et al., 2005	Referral to a Hypertension unit, France, 1975–2003	192	44	89,103	[24]
K.C. Loh et al. 1997	Endocrine clinics, Nova Scotia, Canada, 1986–1995	18	42 (12–81)	3,15	[25]
Charles AG Proye et al., 1994	3 medical centers, France, 1951–1992	310	---	---	[26]
P.F. Plouin et al., 1997	A hospital in Paris, France, 1975–1994	129	42.6 (13–80)	63,66	[27]
Roger R. Perry et al., 1990	Surgery branch of National cancer instate, Maryland, USA, 1982–1989	25 (extra-abdominal and 1 incidental discovery were excluded)	39.6(16–74)	6,19	[28]
TD O’halloran et al., 2001	St. Vincent hospital Ireland, 1950–1997	33	40.6 (12–74)	9,24	[29]
Aguilo et al., 1991	University hospital, Puerto Rico, 1970–1990	24 (19 diagnosed clinically and 5 post-mortem)	43.2 (17–74)	14,10	[30]
J.E. Thomas et al., 1966	Mayo clinic, USA. 1945–1964	100 (97 in life and 3 autopsy)	41 (4–67)	42,58	[31]
Robert Kopetschke et al., 2009	4 endocrine centers, Germany, 1973–2007	183	12–85	---	[32]
C. Charles et al. 1984	A university hospital, Jamaica, 1963–1983	16	38 (13–66)	8,8	[33]
Wei-ber Liao et al., 2000	Chang Gung memorial hospital, Taiwan, 1993–1997	25	49 (16–68)	14-11	[34]
Joyce SY Yau et al. 2010	endocrine clinics of the Kowloon West Cluster hospitals, china. 1994–2003	17 (19 at first. 2 lost to follow up)	47 (17–72)	6,11	[35]
Van Duinen et al. 2010	Leiden university medical center, Netherland, 1975–2008	28 patients whom the pheochromocytoma was diagnosed based on signs and symptoms	47	---	[36]
Bernard Goldny et al., 2001	Department of General Surgery of the University of Munster Hospital, Germany. 1967–1998	133	41.4 (2–75)	68,65	[37]
I.M. Modlin et al. 1979	A number of hospitals, England, 1955–1976	72 (58 diagnosed in life)	---		[38]
E. J. Ross and Griffith 1989	3 hospitals, London, England, 1952–1982	54	12–74	16,38	[39]
Ulf Niemann et al., 2002	University hospital, Germany. 1974–2000	87	49 (14–78)	38,49	[40]
Takyo Noshiro et al., 2000	Tohoku University School of Medicine, Sendai, Japan. 1957–1995	95 (2 autopsy)	---	43,52	[42]
Richard E. Goldstein et al.,1999	2 medical centers, Tennessee, USA, 1950–1998	84	42 (9–79)	38,66	[43]
Khoram manes et al. 2005	Sahlgrenska University Hospital. western region of Sweden. 1950–1997	121	47 (7–71)	53,68	[44]
N. Sharma et al., 2001	Tertiary care hospital, Chandigarh, India, 1989–1996	30	24 (17–31)	17, 13	[45]
Masky P et al. 2012	Tribhuvan University Teaching Hospital, Maharajgunj, Kathmandu, Nepal. 2008–2011	12	36 (12–65)	5,7	[46]

**Table 2:** Studies Assessing Clinical Presentations: studies with control group

Source	Clinical setting, Years	No. of Patients	Age, Mean (Range)	Gender (M, F)	Control group	Ref.
Plouin et al., 1981	Hypertension service, saint-Joseph hospital, Paris, France, from 1977	2585 hypertensive patients (11 of them were found to have pheochromocytoma)	33–60	1443,1142	All of the 2585 patients are considered control group as the proportion of pheochromocytoma is 0.4 %	[13]
Peter P. Stein et al. 1991	Yale university school of medicine, USA.	30 episodes (29 patients), 28 controls	37 (18–65)	13,16	Yes^a^	[16]
			43 (20–65)	16,12		
Henry R. Black et al. 1984	11 new England hospitals, USA, 1962–1980	53 patients (60 first. 5 excluded because of finding based on predisposition genetic factor. 1 excluded because of being asymptomatic and tumor found at autopsy) 25 controls	41 (13–85)	27, 26	Yes^b^	[17]
			39.3 (19–85)	14,11		
P.F. Plouin et al., 1988	Hypertension departments of 2 hospitals, Paris, France, 1976–1985	39 patients	---		Yes (21 patients with essential hypertension)	[20]
		21 controls				
Václavík J et al. 2007	Sternberk Hospital, Sternberk, Czech Republic.	14 patients	58 (37–74)	6,8	Yes^c^	[23]
		214 controls	57 (16–84)	86,128		
Run Yu et al. 2007	An academic hospital, Los Angeles, USA 1997–2007	40 patients	54(10–78)	16,24	Yes (patients with over-diagnosed pheochromocytoma)	[41]
		9 controls	57 (36–82)	2,7		
Yu R et al. 2010	Division of Endocrinology, Cedars-Sinai Medical Center, Los Angeles, California. 2000–2008	13 patients	53 (23–86)	6,7	Yes (24 patients with highly elevated biochemical tests but pheochromocytoma was ruled out)	[47]
		24 controls	59 (28–82)	10,14		

^a^28 (a pheochromocytoma was considered but excluded if any 1 of several conditions were met: 1) repeatedly normal urine collections for catecholamine metabolites (VMA or MN) and urine free catecholamines (UFC) and no diagnosis of pheochromocytoma after 2 years of follow up; 2) negative imaging studies (CT, MRI or MIBG) and no diagnosis of pheochromocytoma after 2 years of follow up; 3) resolution of the clinical symptoms and/or alternate diagnosis, explaining the symptoms, established.)

^b^Patients highly suspected to have pheochromocytoma in whom the diagnosis was ruled out by negative arteriograms and no evidence of disease after at least 18 months follow-up

^c^213 patients screened for resistant or markedly accelerated hypertension, paroxysmal hypertension, and ‘flushes’ and, in a small proportion, for adrenal incidentaloma or genetic predisposition to pheochromocytoma. in who diagnose was not confirmed by long-term follow-up or use of imaging techniques

Based on our definition of heterogeneity, all of our data in groups were heterogenous (except negative LR of hypertension with I^2^ of 43.2 %); so we did meta-analysis with random effect. Number of studies which had reported sensitivity of signs/symptoms, pooled sensitivity with method of random effect and its 95 % confidence intervals are shown in Table 3.

**Table 3:** Sensitivity of signs and symptoms

Sign/symptom	No. of studies report	Pooled sensitivity (Random effects) (%)	95 % CI
Headache	25	60.4	53.2–67.4
Palpitation	19	59.3	51.9–66.6
Diaphoresis	28	52.4	0.457–59.1
Triad	8	58	28.6–84.7
Spells	7	57.5	33.9–79.3
HTN(total)	23	80.7	74.7–85
HTN(sustained)	9	36.3	20.5–53.9
HTN (paroxysmal)	9	36.5	24.6–49.3
HTN (paroxysms on sustained)	4	29.4	17.3–43.1
Chest pain	16	17.3	11.4–24.2
Abdominal pain	11	16.5	11.9–216
Flank pain	2	5.2	20.7–9.6
Dyspnea	10	23.4	16.2–31.5
Anxiety	14	28.6	22.9–34.7
Constipation	4	13.8	32.2–29.9
Diarrhea	2	4	0.8–9.4
Dizziness	11	17.7	13.5–22.3
Flushing	14	15	9.3–21.7
Pallor	7	31.6	17.3–47.9
Nausea/Vomiting	14	21.2	16–26.7
Paresthesia	4	13.6	10–17.8
Tremor	10	20.2	14.5–26.6
Visual disturbance	7	9.6	5.6–14.6
Weakness/Fatigue	8	23.8	15.7–33.9

The definition of orthostatic hypotension was different among studies. The sensitivity based on the definition is shown in Table 4.

**Table 4:** Sensitivity of orthosthatic hypotension based on different definitions in studies

Study (number of patients)	Definition of OH^a^	Sensitivity (%)
M. Mannelli et al. (156)	falling SBP > 30 mmHg or falling DBP > 20 mmHg	23
N. Sharma et al. (30)	fall in SBP > 20 mmHg	50
Baguet et al. [48] (41)	fall in SBP > 20 or fall in DBP > 10 1 min after standing	36.6
Plouin et al. (39)	fall in SBP > 10 mmHg	36

^a^ *OH* Othostatic Hypotension, *SBP* systolic blood pressure, *DBP* diastolic blood pressure

Based on funnel plot and Egger test, paroxysmal hypertension, chest pain, flushing, and weakness were the signs/symptoms which had publication bias.

As we mentioned before, seven of the articles had control group, and therefore could be used for calculating LR of signs/symptoms. Seven of the symptoms were evaluated in these articles: palpitation, diaphoresis, classic triad, hypertension, weakness/fatigue, anxiety and flushing. We draw the 2 × 2 table for each of the symptoms/signs and meta-analyzed the LRs with meta-disc software (Table 5).

**Table 5:** Pooled estimation of LR for symptoms and signs of pheochromocytoma

Sign/symptom	Number of studies	LR+ (95%CI)	LR- (95%CI)
Palpitation	2	1.888 (1.161–3.073)	0.518 (0.333–0.806)
Diaphoresis	2	2.184 (1.411–3.382)	0.451(0.310–0.657)
Classic triad^a^	2	6.312 (0.217–183.217)	0.139 (0.059–0.331)
Hypertension	5	0.762 (0.562–1.033)	1.682 (1.093–2.589) fixed effect
Weakness/fatigue	2	1.123 (0.658–1.919)	0.964 (0.772–1.205)
Anxiety	2	1.127 (0.500–2.541)	0.933 (0.635–1.369)
Headache	2	1.607 (1.124–2.297)	0.240 (0.094–0.613)
Flushing	2	0.283 (0.058–1.391)	1.466 (0.754–2.850)
Spells	1^b^	0.93	1.16
Pallor	1	4.667	0.718
Diarrhea	1	0.311	1.188
Constipation	1	0.156	1.230
Dizziness	1	0.431	1.493
Paresthesia	1	1.867	0.933
Tremor	1	0.560	1.096
Orthostatic hypotension^c^	1	1.885	0.792

^a^In all but classic triad, the control group was highly suspected but ruled out pheochromocytoma. In the classic triad, the control groups were patients with hypertension

^b^These symptoms were evaluated in Stein’s article [16]

^c^The definition of orthostatic hypotension in Plouin study was falling SBP >10 mmHg, and the control group was patients with essential hypertension

**Table:** 

		Pheochromocytoma	
		+	-
Symptom/sign	+	TP	FP
	-	FN	TN

## Discussion

The main purpose of this article was defining sensitivity and – if possible- LR of signs and symptoms in diagnosis of pheochromocytoma. We used the search strategy of “Rational Clinical Examination” for collecting articles in Medline from 1960 to 2014; and used reference tracking of review articles to find more relevant original articles. In addition to electronic search, we did hand-searching using endocrinology textbooks. The result of this search strategy was 37 articles. From 6 of these articles, we could fill the 2 × 2 table of LRs.

For evaluating the possibility of doing meta-analysis for our data, first we calculated heterogeneity of different symptoms, which all were heterogeneous according to definition of heterogeneity based on I^2^ (except negative LR of hypertension with I^2^ of 43.2 %). Because of heterogeneity of data, we used random effect model for meta-analysis. For evaluating publication bias we used Egger test, which the *P*-value < 0.05 was considered significant and so the data was considered to have publication bias. This was true for 4 of the symptoms: paroxysmal hypertension, chest pain, flushing and weakness.

The meta-analysis of positive and negative LRs for symptoms and signs was done. Therefore, **palpitation** with positive LR of 1.888 (95 % CI 1.161–3.073) and negative LR of 0.518 (0.333–0.806), **diaphoresis** with positive LR of 2.184 (1.411–3.382) and negative LR of 0.451(0.310–0.657) and **headache** with positive LR of 1.607 (1.124–2.297) and negative LR of 0.240 (0.094–0.613) were the symptoms useful in differentiating pheochromocytoma from other similar diseases. In addition, not having the classic triad had the LR of 0.139 (0.059–0.331) (Table 5). One was included in confidence interval of a number of LRs, but because of asymmetry of the intervals, the numbers could be considered clinically significant. These, include classic triad with the positive LR of 6.312 (0.217–183.217), hypertension with the positive LR of 0.762 (0.562–1.033), anxiety with the positive LR of 1.127 (0.500–2.541), and flushing with the negative LR of 1.466 (0.754–2.850). Among our data only one feature proved to be homogenous: negative LR of hypertension (LR- of 1.682 with 95 % confidence interval of 1.093–2.589).

Based on Plouin study in 1981 on 2585 hypertensive patients, classic triad of headache, palpitation and diaphoresis has the LR of 14.63 (positive) and 0.1 (negative) in patients with hypertension for diagnosis of pheochromocytoma [13]. We found similar negative LR (0.139) for classic triad but the result of positive LR was different (6.3). When we looked at 2 studies of Plouin for evaluating classic triad in diagnosis of pheochromocytoma, [13, 20] we noticed some differences (Table 6).

**Table 6:** Two studies of P.F. Plouin for evaluating classic triad in diagnosis of pheochromocytoma

Authors, number of patients and control group	TP	FP	FN	TN
P.F. Plouin et al. 1988, *N* = 39/21essential HTN	35	7	4	14
Plouin et al. 198l, *N* = 2585 HTN whose 11 patient had pheochromocytoma	10	160	1	2414

*TP* True Positive, *FP* False Positive, *FN* False Negative, *TN* True Negative

As shown on the table, the most significant difference between 2 studies is the ratio of false negative results to all negative results (4/14 vs. 1/2414). An explanation can be the precedence of the first study (1981) with larger sample size [13]. At that time thinking about the triad symptoms was not routine and evidence-based. So, it is possible that asking about them was not done in a series of patients and this group was classified as “not having the symptoms”. So, the ratio of patients that apparently didn’t have the symptoms and finally diagnosed as “having pheochromocytoma” was increased (As the study was retrospective and based on patients’ files).

Hypertension is the most famous sign among physicians for clinical suspicion of pheochromocytoma. What we found was somehow different. We found positive LR of 0.762 (0.562–1.033) with an asymmetry through below 1 (means that hypertension decreases the probability of pheochromocytoma), and negative LR of 1.682 (1.093–2.589) with heterogeneity below 50 % (means homogeneity of data and so fixed effect meta-analysis), which shows that normotension increases the probability of pheochromocytoma. Our explanation is that may be in patients without hypertension the threshold to think about and refer for further analysis of pheochromocytoma is higher for clinicians, and so more patients finally would be proved having pheochromocytoma.

Some of the symptoms were only reported in Stein’s article [16]. Pallor, dyspnea, paresthesia, and orthostatic hypotension were the symptoms and signs which their presence increases the likelihood of pheochromocytoma; whereas diarrhea, constipation, dizziness, chest pain and tremor were the symptoms and signs which their presence decreases the likelihood of pheochromocytoma. Diarrhea and constipation are non-specific symptoms related to pheochromocytoma; so, their incidence is not valuable accordingly.

The LRs of orthostatic hypotension was studied in Plouin article [20]. The control group were patients with essential hypertension and the definition of orthostatic hypotension was falling systolic blood pressure more than 10 mmHg after standing. The positive LR 1.885(0.710–5.003) and the negative LR 0.792(0.579–1.083) were calculated. The value of triad plus orthostatic hypotension was studied too. When considering this combination, the sensitivity was decreased from 89 % (considering triad alone) to 30 %, and the specificity was increased from 67 to 95 %. (LR + = 6.462(0.901-46.325), LR- = 0.727(0.578–0.915).

By drawing the Funnel plot for each sign/symptom and doing Egger test for assessing publication bias, paroxysmal hypertension, chest pain, flushing and weakness were the signs/symptoms which had publication bias based on our definition. So, these items reasonably should be excluded from our final report.

At last, when we look at the table of LRs, it seems that no single sign or symptom alone is helpful in diagnosis of pheochromocytoma. Rather, the combination (such as the classic triad) can be probably important for this aim. Despite flushing and pallor had significant positive LRs and headache had significant negative LR, these were evaluated in only one study and additional studies should be done for more accuracy. Studying the triad in hypertensive patients decrease the spectrum bias and approximates the LRs to reality in clinical setting. According to Plouin’s study (11 pheochromocytoma in 2585 hypertensive patients), the probability of pheochromocytoma in hypertensive patients with classic triad becomes 2.6 %, compared with 0.4 % in hypertensive patients [13]. By experts’ consensus, this number could be high enough for continuation of evaluating the disease. On the other hand, the probability of pheochromocytoma in hypertensive patients without classic triad becomes 0.05 % (compared with 0.4 % in patients with classic triad). So it seems reasonable to clinically rule out the disease by this data.

### Limitations

The limitations of our study were looking for English articles only, and using only PubMed for our search. For compensation of this limitation, we used reference tracking for expanding the results. This was done till we reached the point that the articles we were finding became duplicated. Also we looked at articles which aimed to diagnose pheochromocytoma biochemically (we hoped to find control groups and therefore could calculate LR).

The other problem was verification bias. Patients with known signs/symptoms are usually referred for evaluation of pheochromocytoma. Patients with less common presentations are less evaluated and this overestimates sensitivity and underestimates specificity of the findings. Because most of the studies were done in referral centers, the verification bias would further increases. Doing the studies in tertiary referral centers also cause spectrum bias, because the prevalence of pheochromocytoma defers from the general population in this setting. In some of the studies found, the prevalence of pheochromocytoma in the population studied was reported. This, has a range of 1.5–6.7 % (compared to prevalence of 1−4 × 10^−6^ in the general population) [21–23].

An important point is the paucity of studies about value of clinical presentation regarding pheochromocytoma. Because of heterogeneity of most of the clinical studies and wide confidence interval of results, additional studies are recommended to narrow the confidence intervals and increase the precision of the results.

### Scenario resolution

#### Case 1

As the essential hypertension is most commonly seen in middle aged population, the presence of paroxysmal hypertension in a young patient leads us to pathological conditions such as pheochromocytoma. The presence of classic triad of headache, palpitation and sweating raises the clinical suspicion 6 times. The other symptoms (dizziness, chest pain, dyspnea) are not independent. So we cannot simply multiply them to calculate the final LR. By considering the value of signs and symptoms, we decided to move forward in our evaluation. The level of urine catecholamines was increased. Imaging showed a 3 cm mass in left adrenal gland. The patient was referred to surgeon for left adrenalectomy. The pathologist reported chromaffin cells and the diagnosis of pheochromocytoma was proved. The attacks of the patient subsided after surgery and the blood pressure became stable during 2 years of follow up.

#### Case 2

The patient was a known hypertensive patient from 5 years ago on anti-hypertensive drugs. Symptoms of emergent hypertension were asked from the patient (chest pain, blurred vision, hematuria, and headache) and a complete physical exam was done. Then, he was given oral anti-hypertensive drugs and observed for 4 h. He was discharged from the Emergency department and was recommended to visit his family physician. The absence of classic triad and other parts of the history made pheochromocytoma less probable.

## Conclusions

By available data, there is no single clinical finding that has significant value in diagnosis or excluding pheochromocytoma. Combination of certain symptoms, signs and para-clinical exams is more valuable for physicians. Further studies should be done, in order to specify the value of clinical findings –alone or in combination- in favor or against the diagnosis of pheochromocytoma more accurately, to help us distinguish patients who require more evaluation, from those who require no further testing. Until that time the process of diagnosis will be based on clinical suspicion and lab tests followed by related imaging.

## Footnotes

1 This strategy was selected based on the “search strategy for the rational clinical examination- David L. Simel, 1995”.

## References

[1] Adler JT (2008). Pheochromocytoma: Current approaches and future directions. Oncologist.

[2] Ilias I, Pacak K (2008). A clinical overview of pheochromocytomas/paragangliomas and carcinoid tumors. Nucl Med Biol.

[3] Lo CY (2000). Adrenal Pheochromocytoma remains a frequently overlooked diagnosis. Am J Surg.

[4] Lenders JW (2005). Pheochromocytoma. Lancet.

[5] McClellan M, Walther á HR, Keiser W (1999). Marston Linehan Pheochromocytoma: evaluation, diagnosis, and treatment. World J Urol.

[6] Mannelli M, Ianni L, Cilotti A, Conti A (1999). Pheochromocytoma in Italy: a multicentric retrospective study. Eur J Endocrinol.

[7] Favia G, Lumachi F, Polistina F, D’Amico DF (1998). Pheochromocytoma, a rare cause of hypertension: long-term follow-up of 55 surgically treated patients. World J Surg.

[8] Stenström G, Ernest I, Tisell LE (1988). Long-term results in 64 patients operated upon for pheochromocytoma. Acta Med Scand.

[9] Sutton MG, Sheps SG, Lie JT (1981). Prevalence of clinically unsuspected pheochromocytoma. Review of a 50-year autopsy series. Mayo Clin Proc.

[10] Benowitz NL (1988). Pheochromocytoma-Recent advances in diagnosis and treatment [Medical Staff Conference]. West J Med.

[11] Zelinka T, Eisenhofer G, Pacak K (2007). Pheochromocytoma as a catecholamine producing tumor: Implications for clinical practice. Stress.

[12] Plouin PF, Degoulet P, Tugayé A, Ducrocq MB, Ménard J (1981). [Screening for phaeochromocytoma: in which hypertensive patients? A semiological study of 2585 patients, including 11 with phaeochromocytoma (author’s transl)]. Nouv Press Med.

[13] Werbel SS, Ober KP (1995). Pheochromocytoma. Update on diagnosis, localization, and management. Med Clin North Am.

[14] Fauci, Braunwald, Kasper, Hauser, Longo, Jameson, Loscalzo. Harrison’s principles of internal medicine. 17th edition. Part 15. Chapter 337. p 2269.

[15] Lance JW, Hinterberger H (1976). Symptoms of pheochromocytoma, with particular reference to headache, correlated with catecholamine production. Arch Neurol.

[16] Stein PP, Black HR (1991). A simplified diagnostic approach to pheochromocytoma. A review of the literature and report of one institution’s experience. Medicine (Baltimore).

[17] Black HR, Bursten SL (1984). A clinical scoring system for detection of patients with pheochromocytomas. Yale J Biol Med.

[18] The fifth report of the Joint National Committee on Detection, Evaluation, and Treatment of High Blood Pressure (JNC V)**.** Arch Intern Med. 1993;153:154–83. 8422206

[19] Lai EW, Perera SM, Havekes B, Timmers HJ, Brouwers FM, McElroy B, Adams KT, Ohta S, Wesley RA, Eisenhofer G, Pacak K (2008). Gender-related differences in the clinical presentation of malignant and benign pheochromocytoma. Endocrine.

[20] Plouin PF, Chatellier G, Rougeot MA, Duclos JM, Pagny JY, Corvol P, Ménard J (1988). Recent developments in pheochromocytoma diagnosis and imaging. Adv Nephrol Necker Hosp.

[21] Patócs A, Karádi E, Tóth M, Varga I, Szücs N, Balogh K, Majnik J, Gláz E, Rácz K (2004). Clinical and biochemical features of sporadic and hereditary phaeochromocytomas: an analysis of 41 cases investigated in a single endocrine centre. Eur J Cancer Prev.

[22] Hernandez FC, Sánchez M, Alvarez A, Díaz J, Pascual R, Pérez M, Tovar I, Martínez P (2000). A five-year report on experience in the detection of pheochromocytoma. Clin Biochem.

[23] Václavík J, Stejskal D, Lacnák B, Lazárová M, Jedelský L, Kadalová L, Janosová M, Frysák Z, Vlcek P (2007). Free plasma metanephrines as a screening test for pheochromocytoma in low-risk patients. J Hypertens.

[24] Loh KC, Shlossberg AH, Abbott EC, Salisbury SR, Tan MH (1997). Pheochromocytoma: a ten-year survey. QJM.

[25] Proye CA, Vix M, Jansson S, Tisell LE, Dralle H, Hiller W (1994). “The pheochromocytoma”: a benign, intra-adrenal, hypertensive, sporadic unilateral tumor. Does it exist?. World J Surg.

[26] Plouin PF, Chatellier G, Fofol I, Corvol P (1997). Tumor recurrence and hypertension persistence after successful pheochromocytoma operation. Hypertension.

[27] Perry RR, Keiser HR, Norton JA, Wall RT, Robertson CN, Travis W, Pass HI, Walther MM, Linehan WM (1990). Surgical management of pheochromocytoma with the use of metyrosine. Ann Surg.

[28] O’Halloran T, McGreal G, McDermott E, O’Higgins N (2001). 47 years of phaeochromocytomas. Ir Med J.

[29] Aguiló F, Tamayo N, Vázquez-Quintana E, Rabell V, Haddock L, Allende M, Pagán H, González A (1991). Pheochromocytoma: a twenty year experience at the University Hospital. P R Health Sci J.

[30] Thomas JE, Rooke ED, Kvale WF (1966). The neurologist’s experience with pheochromocytoma. A review of 100 cases. JAMA.

[31] Kopetschke R, Slisko M, Kilisli A, Tuschy U, Wallaschofski H, Fassnacht M, Ventz M, Beuschlein F, Reincke M, Reisch N, Quinkler M (2009). Frequent incidental discovery of phaeochromocytoma: data from a German cohort of 201 phaeochromocytoma. Eur J Endocrinol.

[32] Charles C, Grell GA, Fletcher PR, Hanchard B (1984). A clinico-pathological study on pheochromocytoma at the University Hospital of the West Indies. West Indian Med J.

[33] Liao W (2000). Cardiovascular Manifestations of Pheochromocytoma. Am J Emerg Med.

[34] Yau JS, Li JK, Tam VH, Fung LM, Yeung CK, Chan KW, Lee KM, Lee KF, Cheung WS, Yeung VT, Yuen YP, Kwan WK (2010). Phaeochromocytoma in the Hong Kong Chinese population. Hong Kong Med J.

[35] van Duinen N, Steenvoorden D, Bonsing BA, Vuyk J, Vriends AH, Jansen JC, Romijn JA, Corssmit EP (2010). Pheochromocytomas detected by biochemical screening in predisposed subjects are associated with lower prevalence of clinical and biochemical manifestations and smaller tumors than pheochromocytomas detected by signs and symptoms. Eur J Endocrinol.

[36] Glodny B, Winde G, Herwig R, Meier A, Kühle C, Cromme S, Vetter H (2001). Clinical differences between benign and malignant pheochromocytomas. Endocr J.

[37] Modlin IM, Farndon JR, Shepherd A, Johnston ID, Kennedy TL, Montgomery DA, Welbourn RB (1979). Pheochromocytomas in 72 patients: clinical and diagnostic features, treatment and long term results. Br J Surg.

[38] Ross EJ, Griffith DNW (1989). The clinical presentation of pheochromocytoma. QJM.

[39] Niemann U, Hiller W, Behrend M (2002). 25 years experience of the surgical treatment of phaeochromocytoma. Eur J Surg.

[40] Noshiro T, Shimizu K, Watanabe T, Akama H, Shibukawa S, Miura W, Ito S, Miura Y (2000). Changes in clinical features and long-term prognosis in patients with pheochromocytoma. Am J Hypertens.

[41] Goldstein RE, O’Neill JA, Holcomb GW, Morgan WM, Neblett WW, Oates JA, Brown N, Nadeau J, Smith B, Page DL, Abumrad NN, Scott HW (1999). Clinical experience over 48 years with pheochromocytoma. Ann Surg.

[42] Khorram-Manesh A, Ahlman H, Nilsson O, Friberg P, Odén A, Stenström G, Hansson G, Stenquist O, Wängberg B, Tisell LE, Jansson S (2005). Long-term outcome of a large series of patients surgically treated for pheochromocytoma. J Intern Med.

[43] Sharma N, Kumari S, Jain S, Varma S (2001). Pheochromocytoma: a 10-year experience in a tertiary care North Indian hospital. Indian Heart J.

[44] Maskey P, Shrestha GK, Luitel BR, Gupta DK, Sidarth, Chalise PR, Sharma UK, Gyawali PR, Joshi BR (2012). Phaeochromocytoma in Nepal--a single centre experience. Kathmandu Univ Med J (KUMJ).

[45] Yu R, Nissen NN, Chopra P, Dhall D, Phillips E, Wei M (2009). Diagnosis and treatment of pheochromocytoma in an academic hospital from 1997 to 2007. Am J Med.

[46] Yu R, Wei M (2010). False positive test results for pheochromocytoma from 2000 to 2008. Exp Clin Endocrinol Diabetes.

[47] Baguet JP, Hammer L, Mazzuco TL, Chabre O, Mallion JM, Sturm N, Chaffanjon P (2004). Circumstances of discovery of pheochromocytoma: a retrospective study of 41 consecutive patients. Eur J Endocrinol.

[48] Amar L, Servais A, Gimenez-Roqueplo AP, Zinzindohoue F, Chatellier G, Plouin PF (2005). Year of diagnosis, features at presentation, and risk of recurrence in patients with pheochromocytoma or secreting paraganglioma. J Clin Endocrinol Metab.

